# Acroframosome-Dependent KIFC1 Facilitates Acrosome Formation during Spermatogenesis in the Caridean Shrimp *Exopalaemon modestus*

**DOI:** 10.1371/journal.pone.0076065

**Published:** 2013-09-30

**Authors:** Cong-Cong Hou, Wan-Xi Yang

**Affiliations:** The Sperm Laboratory, College of Life Sciences, Zhejiang University, Hangzhou, China; University of South Florida College of Medicine, United States of America

## Abstract

**Background:**

Acrosome formation and nuclear shaping are the main events in spermatogenesis. During spermiogenesis in *Exopalaemon modestus*, a unique microtubular structure called the acroframosome (AFS) forms in spermatids. The AFS links to a temporary organelle called the lamellar complex (LCx) leading to the formation of an everted umbrella-shaped acrosome and a dish-shaped nucleus in the mature sperm. These morphological changes require complex cell motility in which the C-terminal kinesin motor protein called KIFC1 is involved. In this study, we demonstrate that KIFC1 moves along the AFS and plays an important role in acrosome formation and nuclear shaping during spermatogenesis in *E. modestus*.

**Methodology/Principal Findings:**

We cloned a 3125 bp complete cDNA of *kifc1* from the testis of *E. modestus* by PCR. The predicted secondary and tertiary structures of *E. modestus* KIFC1 contain three domains: a) the C-terminus, b) the stalk region, and the c) N-terminusl. Semi-quantitative RT-PCR detected the expression of *kifc1* mRNA in different tissues of *E. modestus*. *In situ* hybridization demonstrated the temporal and spatial expression profile of *kifc1* during spermiogenesis. Western blot identified the expression of KIFC1 in different tissues of *E. modestus*, including the testis. Immunofluorescence localized KIFC1, tubulin, GM130, and mitochondria in order to elucidate their role during spermiogenesis in *E. modestus*.

**Conclusion/Significance:**

Our results indicate that KIFC1 transports the Golgi complex, mitochondria, and other cellular components that results in acrosome formation and nuclear shaping in *E. modestus*. The KIFC1 transport function depends upon the microtubular structure called the acroframosome (AFS). This study describes some of the molecular mechanisms involved in the acrosome formation and nuclear shaping in *E. modestus*. In addition, this study may provide a model for studying the molecular mechanisms involved in spermatogenesis in other crustacean species and lead to a better understanding of the fertilization process in crustaceans.

## Introduction

There are three types of motor proteins that convert chemical energy into mechanical energy and show cytoskeleton dependency. Kinesin and dynein show microtubule dependency and participate in morphological changes and organelle/vesicle transport [1]–[4]. Myosin shows microfilament dependency and participates in various processes like muscle contraction. Kinesin motor proteins use energy released by ATP hydrolysis for organelle/vesicle transport [5]. The molecular mechanisms used by various kinesin motor proteins in morphological changes and organelle/vesicle transport have been elucidated [1], [3].

Neurons also undergo morphological changes during development similar to the morphological changes during spermatogenesis [1], [6], [7]. In this regard, the KIF17 neuron-specific motor protein transports neurosecretory vesicles containing the N-methyl-D-aspartate (NMDA) receptor 2B (NR2B subunit) along microtubules during neuronal morphogenesis [8]. The KIF5 motor protein transports neurosecretory vesicles along axonal microtubules [9]. The kinesin-3 motor protein transports neurosecretory vesicles in a fast anterograde fashion [10].

In addition, KIF1C and kinesin II associate with the Golgi complex and transport Golgi vesicles to the endoplasmic reticulum in a retrograde fashion [11], [12]. The kinesin II motor protein interacts with the intra-flagellar transport complex and functions in mammalian intra-flagellar transport, including the testis [13]. Kinesin transports a unique oligomeric tubulin complex during giant squid neuron development [14], [15]. The KIF5 motor protein associates with a peripheral membrane protein called Gadkin and the clathrin adaptor AP-1 protein and transports endosomal vesicles [16].

Although there are numerous kinesin motor proteins involved in spermatogenesis [17]–[24], we focused on the C-terminal KIFC1 motor protein which belongs to the kinesin-14 subfamily with a highly conserved motor domain [25]. KIFC1 associated with the nuclear membrane participates in acrosome formation via vesicle transport along the outer face of the machete [25], [26]. KIFC1 also transports other cellular components during spermatogenesis in various species [27]–[31]. For example, the KIFC1 motor protein participates in acrosome formation in *Eriocheir sinensis* and cell morphological changes in *Octopus tankahkeei* [27]–[29]. The KIFC1 motor protein also drives acrosome formation and cell morphological changes by interacting with the AFS and LCx during spermatogenesis in *Macrobrachium nipponense* [30]. However, the relationship between the KIFC1 motor protein and the AFS during spermatogenesis in caridean shrimp remains unclear. In order to explore this relationship between KIFC1 and the AFS, we chose the caridean shrimp *Exopalaemon modestus* as our animal model.

There are three events in mammalian spermatogenesis which include: a) acrosome formation, b) nuclear shaping, and c) flagellum formation. However, there are only two events in decapod crustacean spermatogenesis which include only acrosome formation and nuclear shaping because mature crustacean sperm lack flagella [32]–[34]. The two events in decapod crustacean spermatogenesis generally result in the formation of a unique shaped acrosome and nucleus. For example, an intricately-shaped acrosome and a cup-shaped nucleus form in *Eriocheir sinensis* mature sperm [32], [33]. An everted umbrella-shaped acrosome and a dish-shaped nucleus form in the caridean shrimp *M. nipponense* mature sperm [34]–[36]. The morphological changes of caridean shrimp spermatids have been reported in our previous publication [34]–[37]. Briefly, at the early stage, the nucleus firstly is changed from spheroidicity to oblate and the cytoplasmic components began to accumulate at one side of the spermatid [34]. At the middle stage, the main cytoplasmic component is a temporary specific organelle LCx, which primarily contained Golgi apparatus, endoplasmic reticulum, mitochondria, lysosome and centriole [35], [36]. Simultaneously, centriole drives the formation of the microtubular structure AFS, which extends around continuously and provides motor proteins as a support of movement [37]. In particularly, the center of AFS will form a protuberance which is perpendicular to the nucleus and the protuberance grows as a long spike, and the LCx is condensed, compacted and form the acrosome eventually.

Even though the morphological changes that occur during spermatogenesis in caridean shrimp have been reported, the mechanism of acrosome formation in caridean shrimp remains largely unclear at this time [34]. With regards to acrosome formation in caridean shrimp, the LCx and AFS are two important structures that deserve investigation. The temporary organelle called the LCx was first discovered in *M. nipponensis*. The LCx components consist of the Golgi complex, endoplasmic reticulum, mitochondria, lysosomes, and centriole [36]. All of these organelles pull and pack together until an everted umbrella-shaped acrosome forms during the middle stage of spermatid formation. The microtubular structure called the AFS may participate in the pulling and packing together of the above-mentioned organelles that form the LCx. The AFS may originate from the centriole and provide the framework for acrosome formation [37]. The AFS may also provide the framework that holds the LCx together, participate in the accumulation of acrosomal hydrolytic enzymes, maintain the acrosome structure, and facilitate the entry of the sperm nucleus into the ovum during fertilization [30].

Caridean shrimp of different genera share similar processes of spermiogenesis [30], [34]–[37]. We previously reported that the morphological changes during spermatogenesis in *E. modestus* are similar to those in *M. nipponensis* [35], [36]. Therefore, we hypothesize that the AFS may play a similar function during spermiogenesis in *E. modestus* as it does in *M. nipponensis*. This hypothesis raises some interesting questions. Is the AFS a ubiquitous microtubular structure in all caridean shrimp? Do other motor proteins “walk” along the AFS and contribute to the formation of the everted umbrella-shaped acrosome? In this study, we report that the KIFC1 motor protein carries and transports the LCx components along the microtubules of AFS which thereby facilitates acrosome formation during spermiogenesis in *E. modestus*.

## Materials and Methods

### Animals and Sampling

The wild adult *E. modestus* shrimp were purchased from Taihu Lake at Huzhou city in Zhejiang Province (China). We detached the zoetic individuals and collected the tissues such as testis, heart, muscle, hepatopancreas, and gill. The detached tissues were put into liquid nitrogen immediately for RNA and protein extraction. In addition, the testis was fixed with 4% paraformaldehyde in phosphate buffered saline (PBS; pH 7.4) for *in situ* hybridization and immunofluorescence.

No approval for experimentation on caridean shrimp is needed in China.

### RNA extraction and reverse transcription

The Phase Lock Gel™ Heavy with Trizol A^+^ reagent (Tiangen Biotech, Beijing, China) was used to extract total RNA from the testis, heart, muscle, hepatopancreas, and gill. The tissue samples were dissolved and homogenized in Trizol A^+^. The homogenate was transferred to Phase Lock Gel™ Heavy and then treated with chloroform, isopropanol, and 75% ethanol sequentially to get precipitated RNA. The precipitated RNA was redissolved in DEPC-H_2_O and the RNA concentration was measured spectrophotometry. The resuspended RNA was stored at −80°C for subsequent reverse transcription methodology.

The PrimeScript® RT reagent Kit (Takara, Dalian, China) was used for ordinary reverse transcription assays. The Smart RACE cDNA Amplification Kit (CloneTech, Mountain View, USA) was used for 5′ RACE and 3′ RACE reverse transcription assays. The reverse transcription products were stored at −20°C for future PCR.

### Full-length cDNA cloning

We designed the primers (Table 1) in reference to the *kifc1* sequences of *M. rosenbergii* and *M. nipponensis by* using the Primer Premier 5 software in order to get the intermediate segment sequence of *kifc1* in the testis. All the primers were synthesized by Beijing Genomics Institute (BGI) and used to amplify the intermediate segment sequence by Touchdown PCR (TD-PCR). We ran the touchdown PCR programs as follows: 94°C for 4 min; 32 cycles of 94°C for 30 s, 60°C for 30 s, and 72°C for 30 s; and, 72°C for 10 min for the final extension. The PCR products were separated by agarose electrophoresis and the bands visualized by DNA gel green. The expected bands were extracted and purified using AxyPrep DNA Gel Extraction Kit (Axygen, Silicon Valley, USA) and AxyPrep PCR Cleanup Kit (Axygen, Silicon Valley, USA). The purified fragments were ligated to PMD-18T vector (Takara, Dalian, China), propagated into competent cells (*Escherichia coli* DH5α), and finally sequenced by Biosune Company (Shanghai, China).

**Table 1:** The list of all primers used in the study.

Primer name	Sequence	Purpose
F1	GAGAAAAGAACAAGATGAACGA	PCR
R1	GCCCATAGGCAAATATACAGAC	PCR
3′EM-K1-F1	TCTGGTCTTAAAAGTCGAGGAAA	3′ RACE
3′EM-K1-F3	AATTTCACAGCTCGCTCAGAG	3′ RACE
5′EM-K1-R1	TTCTTTTCAGGGCATCGTTCATCTTG	5′ RACE
5′EM-K1-R2	CCAGGTTGGATTTGGAGAACGTGAGA	5′ RACE
β-actinF	TTATGTCGGTGATGAGGC	Positive control of Semi-quantitative RT-PCR
β-actinR	CTGGGGTGTTGAAGGTCT	Positive control of Semi-quantitative RT-PCR
EM-S-F	AAGAAAGGAACAAGATGAACGA	In situ hybridization and Semi-quantitative RT-PCR
EM-S-R	GCCCATAGGCAAATATACAGAC	In situ hybridization and Semi-quantitative RT-PCR

The Smart RACE cDNA Amplification Kit (CloneTech, Mountain View, California, USA) and 3′ full RACE Amplification Kit (Takara, Dalian, China) was used for 3′ and 5′ rapid-amplification of the cDNA ends (RACE) in order to obtain the residual sequence of *kifc1* in the testis. The specific primers (GSPs) for 3′ and 5′ RACE were designed by using the software Primer Premier 5 (Table 1) based on the RACE kit instructions. In addition, several primers supplied by the RACE kits were used with the GSPs. We ran the 3′ RACE programs as follows: 94°C for 4 min; 6 cycles of 94°C for 30 s, 55°C (decreased by 0.5°C/cycle) for 40 s, and 72°C for 1 min; then 33 cycles of 94°C for 30 s, 52°C for 40 s, and 72°C for 1 min; 72°C for 10 min for the final extension. We ran the 5′ RACE programs as follows: 94°C for 4 min; 8 cycles of 94°C for 30 s, 65°C for 40 s, and 72°C for 40 s; then 32 cycles of 94°C for 30 s, 61°C for 40 s, and 72°C for 40 s; 72°C for 10 min for the final extension. The PCR products then underwent extraction, purification, and sequencing of the target fragments as described above.

### Sequence analysis and phylogenetic analysis

BLAST online provided by the National Center for Biotechnology Information (NCBI) (http://www.ncbi.nlm.nih.gov/) was used to analyze the similarity between the *kifc1* cDNA/protein sequences and the known sequences. The Vector NT110 (Invitrogen, California, USA) software was used to align multiple sequences of KIFC1 proteins for analysis of conserved sequences. The Mega4 (version 5.0) software was used to construct the phylogenetic tree by the neighbor-joining method. The KIFC1 homologues in different species were used for comparison and construction of the phylogenetic tree. The Genbank accession numbers were as follows: *Eriocheir sinensis* (GU990077.1), *Gallus gallus* (NM_001081698.1), *Xenopus laevis* (NM_001087534.1), *Homo sapiens* (NM_002263.3), *Danio rerio* (NM_131206.1), *Salmo salar* (ABQ59663.1), *Rattus norvegicus* (NP_001005878.1), *Cynops orientalis* (ADM53352.1). PROSITE (http://prosite.expasy.org/) and Mobyle (http://mobyle.pasteur.fr/cgi-bin/portal.py) was used to predict the secondary structure of the KIFC1 protein. I-TASSER (http://zhanglab.ccmb.med.umich.edu/I-TASSER) was used to predict the 3-D structure of the KIFC1 protein [38]–[40].

### Semi-quantitative RT-PCR analysis of *kifc1* mRNA expression in different tissues

The PrimeScript® RT reagent Kit (Takara, Dalian, China) was used for the reverse transcription assay of different tissues. The Primer Premier 5 software was used to design two specific primers (Table 1) for the analysis of *kifc1* mRNA expression in different tissues. Two specific primers of *actin* (Table 1) were used to amplify a β-actin cDNA fragment for positive control. PCR was run as follows: 94°C for 4 min; 35 cycles of 94°C for 30 s, 60°C for 30 s, and 72°C for 30 s; 72°C for 10 min for the final extension. All PCR products were detected by agar gel electrophoresis. The Quantity 1 (version 4.4.0) software from Tanon Science & Technology Co., Ltd was used to analyze the relative expression quantities in different tissues.

### *In situ* hybridization (ISH)

#### Tissue preparation

The testes of the caridean shrimp were embedded into an optimum cutting temperature (O.C.T) compound and sectioned at 6 µm thickness using a sliding microtome. The tissue sections were mounted on RNase-free poly-l-lysine-coated slides and the samples were rapidly stored at −80°C.

#### Riboprobe synthesis

wo special primers were designed and used to amplify the target cDNA fragment (662 bp) for riboprobe synthesis. The PCR was run as follows: 94°C for 4 min; 35 cycles of 94°C for 30 s, 60°C for 30 s, and 72°C for 40 s; 72°C for 10 min for the final extension. The target cDNA products were sequentially inserted into the PGEM-T EASY Vector (Promega, Beijing, China), propagated into competent cells (Escherichia coli DH5a), and sequenced by Biosune Company (Shanghai, China). The plasmid with 662 bp nucleotides of *kifc1* was linearized with incision enzyme and transcribed *in vitro* with T7 promoter. The riboprobes were precipitated with ethanol and LiCl and were resuspended in DEPC-treated H_2_O. The riboprobe concentration and quality were assessed by spectrophotometer and nucleic acid electrophoresis, respectively.

#### Prehybridization and hybridization

The testis sections were set at room temperature for 10 min and fixed with 4% paraformaldehyde (PFA, pH 7.4) for 10 min. Subsequently, the sections were rinsed for 10 min in 0.1% diethylpyrocarbonate (DEPC)-activated 0.1 M phosphate-buffered saline (PBS, pH 7.4) at room temperature twice and equilibrated for 15 min in 5×SSC (sodium chloride 0.75 M, sodium citrate 0.075 M, pH 7.0). Then the prehybridization buffer consisting of 50% deionized formamide, 40 µg/mL denatured salmon sperm DNA, and 5×SSC solution was placed on the sections for 2 h at 55°C. The hybridization buffer consisting of approximately 300 ng/mL of denatured and digoxigenin (DIG) -labeled riboprobes was placed on the sections overnight at 57°C. Then, the sections were sequentially rinsed for 30 min in 2×SSC solution (pH 7.0) at room temperature; 1 h in 2×SSC solution (pH 7.0) at 65°C water bath; and 1 h in 0.1×SSC solution (pH 7.0) at 65°C water bath.

#### Detection of the product signal

After equilibrating for 5 min in Buffer I (0.1 M Tris–hydrochloride, 0.15 M NaCl, pH 7.5), the sections were incubated for 2 h at room temperature in DIG Buffer I consisting of 1∶4000 anti-DIG alkaline phosphatase conjugated Fab fragments (Roche, Branford, USA) and 0.5% (w/v) blocking reagent (Roche, Branford, USA). Then, the sections were washed three times (each for 15 min) in Buffer I and equilibrated for 5 min in Buffer II (0.1 M Tris–HCl, 0.1 M NaCl, 0.05 M MgCl2, pH 9.5). The chromogenic agent (330 µg/mL nitroblue tetrazolium chlorideand (NBT) and 165 µg/mL 5-bromo-4-chloro-3-indolyl-phosphate (BCIP) in Buffer II) (Promega, Beijing, China) was added onto the sections for coloration in the dark for 2 hours at room temperature. The reaction was terminated by rinsing the sections with 10 min in TE buffer (10 mM Tris–HCl, 1 mM EDTA, pH 8.0). The sections were washed in 95% ethanol for 2 hours in order to remove any non-specific staining. The sections were washed in deionized water for 15 min in order to remove any precipitated Tris crystals. The sections were dehydrated with 50, 75, 95, and 100% ethanol in succession, each for 15 min. Finally, the sections were infiltrated in xylol, mounted in neutral resin, and cover slipped. The hybridized sections were observed by using a Nikon Eclipse E80i microscope (Nikon, Tokyo, Japan) under brightfield illumination. The negative control was performed without adding any probes (data is not shown).

### Prokaryotic expression

Sequences encoding *kifc1* were amplified by PCR with primers (Table 1) containing a BamHI site added on the 5′ end and a EcoRI site added on the 3′ end. The resulting amplifications of *kifc1* sequence were digested and ligated into pET28a (Invitrogen Life Technologies, California, USA). The recombinant plasmid DNAs were transformed into Rosetta (DE3) pLysS (Novagen, Darmstadt, Germany). A single colony of Rosetta harboring the recombinant plasmids was inoculated into 300 ml of Luria-Bertani medium containing chloramphenicol (100 mg/l) with kanamycin (25 mg/l). The culture was shaken at 220 rpm and incubated at 37°C until the OD_600_ value reached 0.8. Isopropyl-b-D-thiogalactoside was added to a final concentration of 1 mM and the culture was shaken continually at 220 rpm and incubated at 37°C for 10 h. After ultrasonication, the precipitates were resuspended in 8 M urea. Recombinant HIS-KIFC1 protein in the resuspended liquids were purified by nickel-nitrilotriacetic acid agarose affinity chromatography according to the QIA expressionist manual (Qiagen, Frankfurt, Germany) and assessed by 12% SDS-PAGE. The purified recombinant HIS-KIFC1 protein was used to make antibody in Hangzhou HuaAn Biotechnology Co., Ltd. (Hangzhou, China).

### Antibodies

The KIFC1 polyclonal antibody was prepared by Hangzhou HuaAn Biotechnology Co., Ltd. (China). Rabbit anti-β-actin was purchased from BIOS (Shanghai, China). FITC conjugated monoclonal anti-tubulin was purchased from Sigma (St. Louis, Mo., USA). HRP conjugated goat anti-rabbit IgG was purchased from Immunology Consultants Laboratory, Inc. Texas Red-conjugated affinipure goat anti-rabbit IgG was purchased from Protein Tech Group, Inc. The Mito-Tracker Green FM was purchased from Invitrogen (California, USA).

### Western blot analysis

Different tissues from adult *E. modestus* were homogenized in RIPA Lysis Buffer (Solarbio, Shanghai, China) containing protease inhibitors. The homogenates were centrifuged at (14,000 rpm) at 4°C for 15 min and the supernatant liquids were collected. The protein concentrations were detected by the Coomassie brilliant blue method and the protein samples were dissolved in 5*SDS sample buffer. The protein samples with equal amounts of protein were separated on 12% gels (SDS-PAGE) and transferred onto the PVDF membrane (Bio-Rad, California, USA). The membrane was blocked with 5% BSA in PBST (PBS with 0.5% Tween 20) for 1 h. Then, the membrane was incubated overnight at 4°C with rabbit anti-KIFC1 antibody (diluted 1∶500) in 5% BSA. After washing three times with PBST for 1 h, the membrane was incubated for 1 h with secondary antibody HRP-conjugated goat anti-rabbit IgG (diluted 1∶1000) in 5% BSA. After that, the membrane was washed three times with PBST for 45 min, three times with PBS for 30 min and three times with ultrapure water for 15 min. The membrane was developed according to the manufacturer's instructions of SuperSignal West PicoTrial Kit (Thermo, Massachusetts, USA). The emitted light was detected using a chemiluminescence imaging (Chemiscope 3400) (Shanghai, China).

### Immunofluorescence staining

The testes of adult male *E. modestus* were fixed in 4% paraformaldehyde (PFA, pH 7.4) overnight, rinsed in PBS three times for 15 min, and incubated overnight in 0.5 M sucrose in PBS. The testes were embedded in Tissue-Tek O.C.T. Compound. The tissue blocks were stored in the refrigerator (−20°C) and cut into 6-mm frozen sections using a cryostat. The sections were washed with PBST (PBS with 0.3% Triton X-100) for 15 min at room temperature and then blocked with 5% BSA in PBST (0.1% Triton X-100) for 1 hour. The tissue sections were incubated with anti-KIFC1 antibodies (1∶80 dilution) in blocking buffer 5% BSA at 4°C overnight. The sections were rinsed 3 times in PBST for 45 min. The tissue sections were then incubated with Texas Red goat anti-rabbit IgG (1∶100 dilution, Jackson Immuno Research Laboratories, West Grove, PA., USA) or FITC-anti-Tubulin conjugated goat anti-mouse IgG (Sigma, St. Louis, MO, USA) secondary antibodies for 1 hour at room temperature. The sections were rinsed 3 times in PBST for another 45 min. The nuclei were stained with DAPI (Beyotime, Dalian, China). The immunostained tissues sections were mounted in Antifade Mounting Medium (Vectashield, Vector Laboratories). The immunostained tissue sections were observed with a Confocal Laser-scanning Microscope (CLSM 510) (Carl Zeiss, Germany).

### Electron Microscopy

Sample for transmission electron microscopy (TEM) observation was performed according to our previous publications [27.28]. Briefly, SD rat testis were prefixed with 2.5% glutaraldehyde, cut into 1 mm^3^ in size, then fixed in the same fixative at 4°C for 2 hours, and then the samples were post-fixed in 1% osmium tetroxide solution (Electron Microscopy Sciences, Hatfield, PA, USA) at room temperature for 1 hour. Samples were washed twice with sodium phosphate buffer. Subsequently, the samples were prestained with uranyl acetate and dehydrated in graded alcohol and acetone. The Spurr's resin was used to embed the samples. The samples were sectioned with a Leica Ultracut UC6 Ultramicrotome (Leica Microsystems AG, Wetzlar, Germany), counterstained with uranyl acetate and lead citrate. Finally, PHILIPS TECNAI 10 (PhilipsMedical System, Best, Netherlands) TEM operated was used to observe the grids at 80 kV [25].

## Results

### *kifc1* sequence analysis

**Figure 1:**
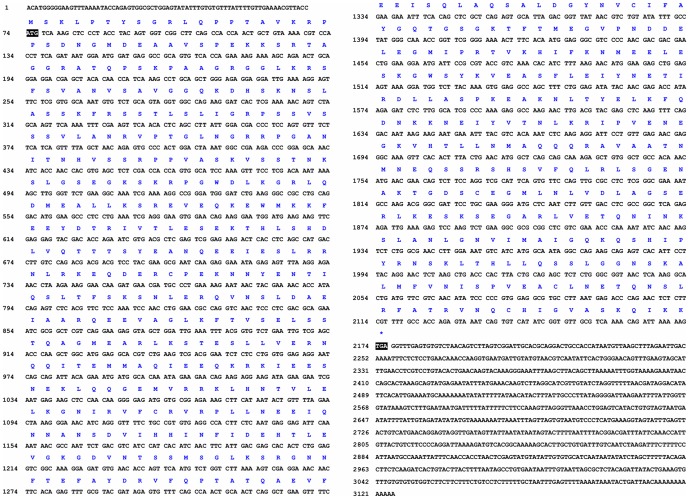
Full-length cDNA of the *kifc1* in *E. modestus*. The deduced amino acid sequence is shown above the nucleotide sequence. This figure shows the full-length cDNA of the *kifc1* that consists of a 73 bp 5′untranslated region, a 952 bp 3′untranslated region and a 2100 bp open reading frame that encodes 700 amino acids.

We used a pair of primitive primers to obtain a 662 bp middle fragment. Subsequently, we designed special primers to amplify a 797 bp sequence and a 1787 by sequence for 5′ RACE and 3′ RACE, respectively. The full-length cDNA of the *kifc1* in *E. modestus* consists of a 73 bp 5′untranslated region, a 952 bp 3′untranslated region, and a 2100 bp open reading frame that encodes 700 amino acids (Fig. 1).

### KIFC1 protein sequence alignment and phylogenetic analysis

**Figure 2:**
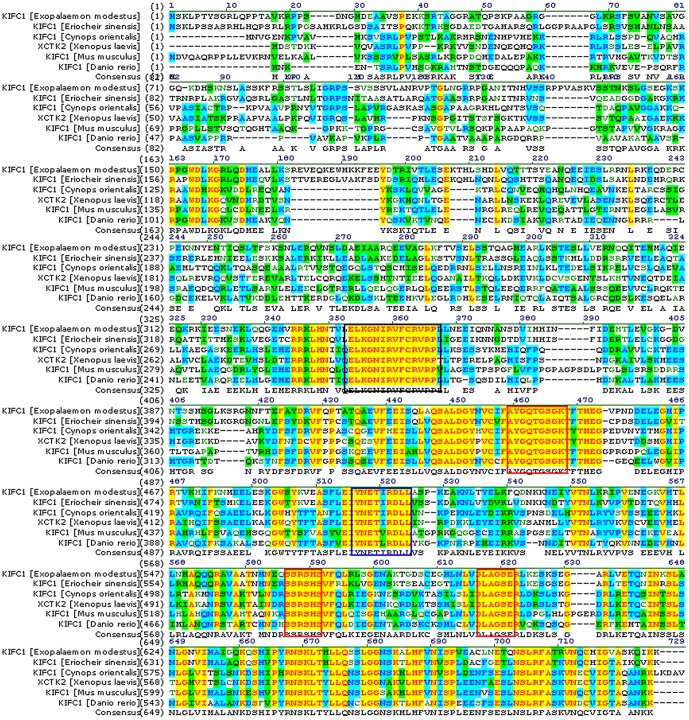
Comparison of the KIFC1 protein in *E. modestus* with its homologues. This figure shows the amino acid alignment of KIFC1 with its homologues using Vector NTI10 (Invitrogen, California, USA). The AYGQTGSGKT, SSRSH, and LAGSE sequences (red frame) are the putative ATP-binding motifs. The YNETIRDLL sequence (blue frame) is the microtubule-binding motif. The ELKGNIRVFCRVRP sequence (black frame) is the KIFC conserved consensus. The sequenced KIFC1 shows a 69.9, 47.8, 47.9, 49.2, and 46.9% identity with its counterparts in *Eriocheir sinensis*, *Crassostrea gigas*, *Xenopus laevis*, *Mus musculus*, and *Danio rerio*, respectively.

**Figure 3:**
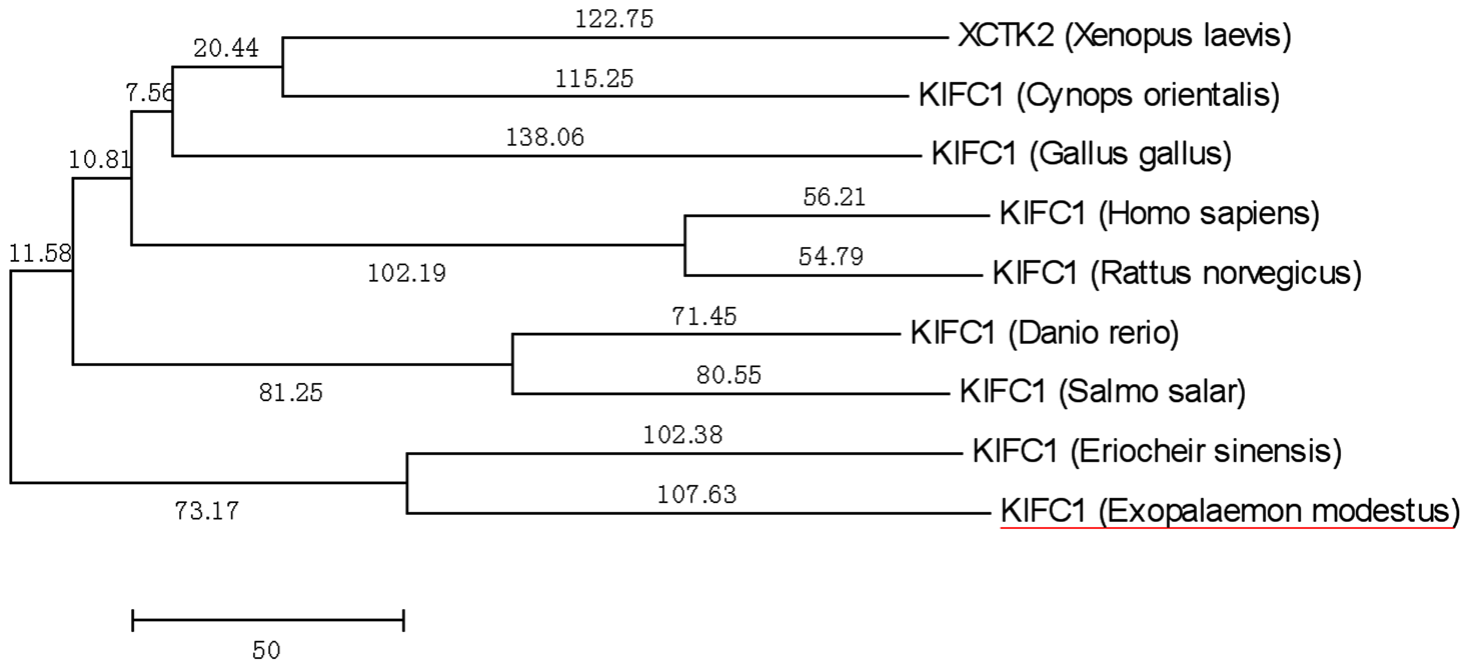
The phylogenetic tree of KIFC1 protein and its homologues. This figure shows the phylogenetic tree of KIFC1 and it homologues that were constructed by the neighbor-joining method using Mega 5 (version 5.0) software. The KIFC1 homologues from *Eriocheir sinensis*, *Gallus gallus*, *Xenopus laevis*, *Homo sapiens*, *Danio rerio*, *Salmo salar*, *Rattus norvegicus*, and *Cynops orientalis were examined*. The putative *E. modestus* KIFC1 protein is most closely related to *Eriocheir sinensis*.

The alignment of KIFC1 and its homologues in different species shows that the sequenced KIFC1 has 69.9, 47.8, 47.9, 49.2, and 46.9% identity with its counterparts in *Eriocheir sinensis*, *Crassostrea gigas*, *Xenopus laevis*, *Mus musculus*, and *Danio rerio*, respectively (Fig. 2). Phylogenetic analysis demonstrates that the putative protein of KIFC1 is closer to *Eriocheir sinensis* than other examined species (Fig. 3).

### KIFC1 protein structure

**Figure 4:**
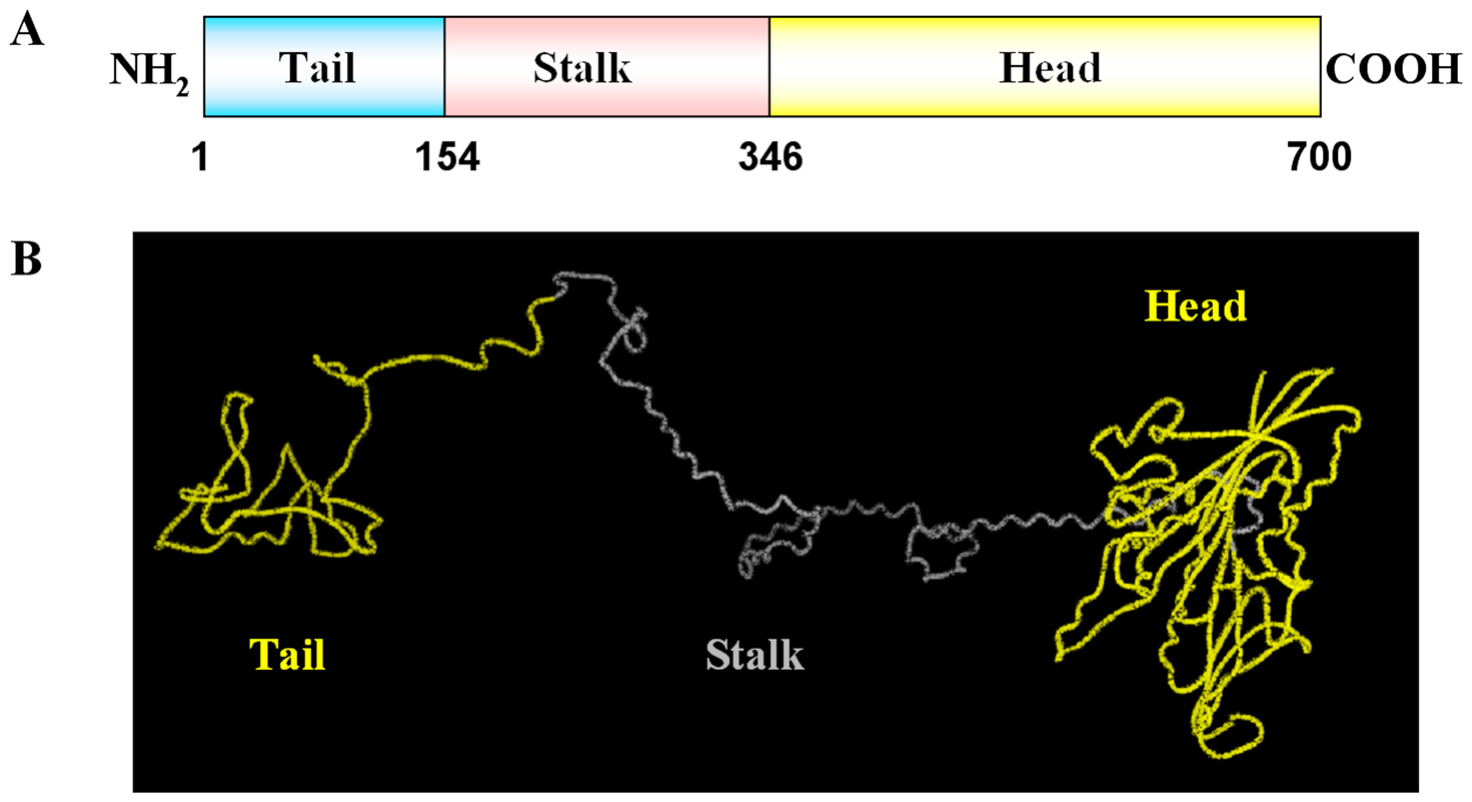
The major structural features of *E. modestus* KIFC1. (A) This figure shows the three structural domains of KIFC1. The C-terminus (346–700 aa) contains the conserved head (yellow bar) that “walks” along the microtubules. The stalk region (154–346 aa) forms an extended coiled-coil region (pink bar). The N-terminus (1–154 aa) contains the divergent tail (blue bar) that carries different cargoes. (B) This figure shows the putative 3-D structure of KIFC1. The head, stalk, and tail are labelled by different colors. See the website http://zhanglab.ccmb.med.umich.edu/I-TASSER/output/S123537/ for more details.

The predicted secondary structure of the *E. modestus* KIFC1 motor protein contains three domains: a) the C-terminus, b) the stalk region, and c) the N-terminus (Fig. 4A). The 346–700 amino acids constitute the putative C-terminus and contain the conserved motor domain that forms a head for “walking” along the microtubule (Fig. 4A). The 154–346 amino acids constitute the coiled stalk region. The 1–154 amino acids constitute the N-terminus with a divergent tail to carry different cargoes (Fig. 4A). The tertiary structure of KIFC1 motor protein comports with the secondary structure characteristics (Fig. 4B).

### *kifc1* mRNA expression in different tissues

**Figure 5:**
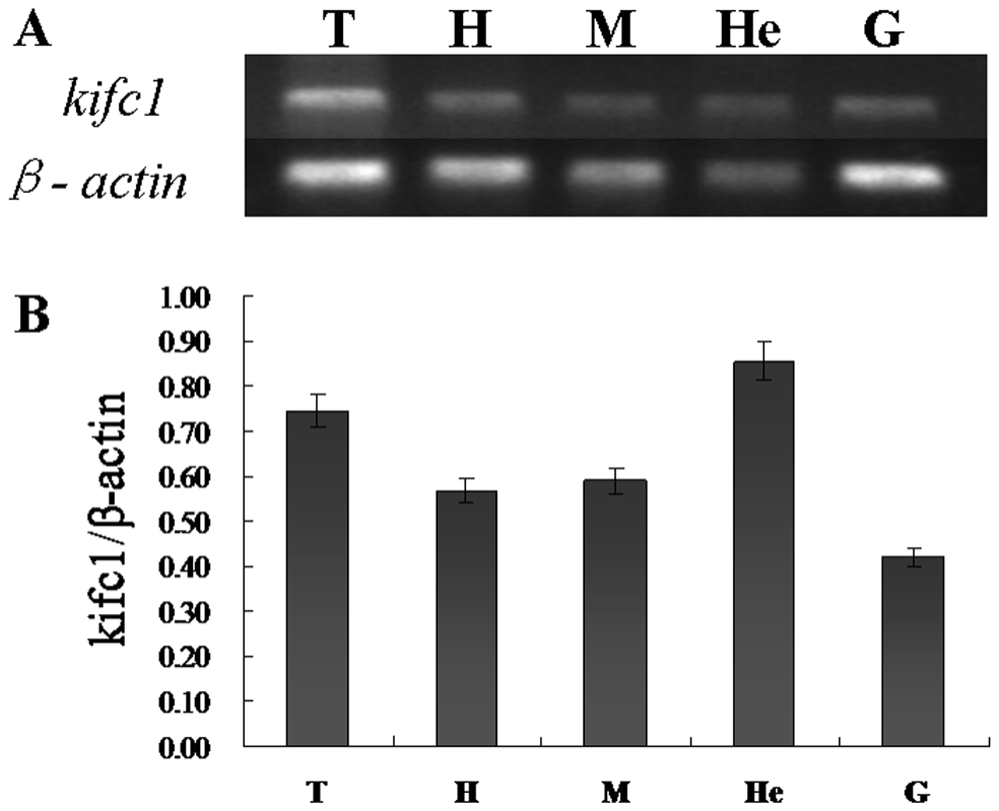
Semi-quantitative RT-PCR analysis of *kifc1* gene in different tissues. (A) This figure shows the *kifc1* expression in various *E. modestus* tissues (upper panel). β-actin was used as a positive control (lower panel). *kifc1* is highly expressed in the *E. modestus* testis. (B) This figure shows a quantitative analysis of *kifc1* expression in various *E. modestus* tissues. *kifc1* is highly expressed in the *E. modestus* testis and hepatopancreas. T: testis, H: heart, M: muscle, He: hepatopancreas and G: gill.

We used semi-quantitative RT-PCR to detect *Kifc1* mRNA expression in various tissues of *E. modestus*. We amplified a 662 bp *kifc1* fragment from the testis, heart, muscle, hepatopancreas, and gill (Fig. 5A). A 297 bp *β-actin* fragment was used as positive control (Fig. 5A). We found that *kifc1* mRNA expression occurs abundantly in all of the above-mentioned tissues, including the testis (Fig. 5B).

### Temporal and spatial expression pattern of *kifc1* mRNA during spermiogenesis

**Figure 6:**
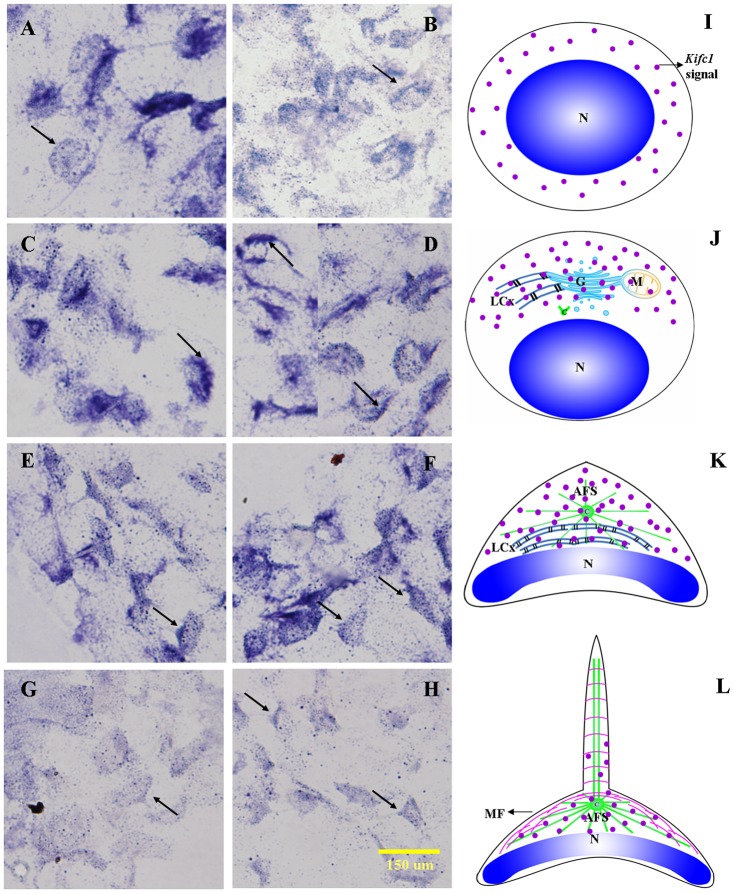
*In situ* hybridization of *kifc1* mRNA during the *E. modestus* spermiogenesis. (A,B,I) Early stage of spermiogenesis. These figures show that *kifc1* mRNA signals (arrows; blue signal; red dots) are weakly distributed in the cytoplasm of round or oblong spermatids. (C,D,J) Middle stage of spermiogenesis. These figures show that *kifc1* mRNA signals (arrows; blue signal; red dots) are increased compared to the early stage and concentrate on one side of the spermatid where the acrosome will eventually form. (E,F,K) Late stage of spermiogenesis. These figures show that *kifc1* mRNA signals (arrows; blue signal; red dots) are strongly distributed in the cytoplasm, centralized over the acrosome cap, and localized in a dot-like pattern on the surface of the dish-shaped nucleus. The AFS-derived spike shows a minimal signal. (G,H,L) Mature sperm. These figures show that *kifc1* mRNA signals (arrows; blue signal; red dots) are weakly distributed over the acrosome cap but, in general, are dramatically decreased compared to other stages of spermiogenesis. The AFS-derived spike and the dish-shaped nucleus show minimal signal. N: nucleus, M: mitochondria, G: Golgi complex, LCX: lamellar complex, AFS: acroframosome, MF: microfilaments.

We used *in situ* hybridization to track the temporal and spatial expression patterns of *kifc1* mRNA. We found that the localization of *kifc1* mRNA closely relates to acrosome formation (Fig. 6). During the early stage of spermiogenesis, *kifc1* mRNA signals weakly distribute throughout the cytoplasm in round or oblong spermatids (Fig. 6A and B, arrows; blue signal). Later in spermiogenesis, the cytoplasmic components gather to one side of the spermatid and the LCx gradually develops. At this time, *kifc1* mRNA signals increase compared to the early stage and concentrate on the side of the spermatid where the acrosome will eventually form during the middle stage of spermiogenesis (Fig. 6C and D, arrows). During the late stage of spermiogenesis, the AFS appears and the nucleus becomes dish-shaped. In addition, *kifc1* mRNA signals strongly distribute at the acrosome cap, display a dot-like pattern on the surface of the dish-shaped nucleus, and are absent at the AFS-derived spike (Fig. 6E and F, arrows). In the mature sperm, kifc1 mRNA signals dramatically decrease in general. The kifc1 mRNA signals weakly distribute at the acrosome cap, are absent at the dish-like nucleus, and are absent at the AFS-derived spike (Fig. 6G ad H, arrows). A series of schematic diagrams clarify the temporal and spatial kifc1 mRNA expression patterns during spermiogenesis (Fig 6I, J, K, and L).

### Identification of KIFC1 protein in various tissues of *E. modestus*

**Figure 7:**
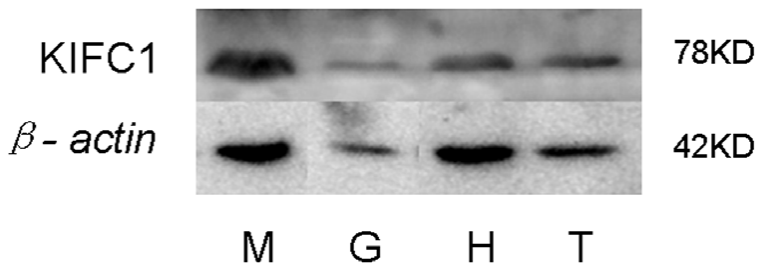
Western blot analysis of *E. modestus* tissues. The *E. modestus* tissue extracts were probed with anti-KIFC1 polyclonal antibody (upper panel) and anti-β-actin polyclonal antibody (lower panel) which served as the control. This figure shows that KIFC1 protein is present at the highest levels in the muscle (M), intermediate levels in the testis (T) and heart (H), and the lowest level in the gill (G). The molecular weight of KIFC1 (78 kDa) and β-actin (42 kDa) is shown at the right.

We used Western blots to identify KIFC1 protein expression in *E. modestus* muscle, gill, heart and testis using an anti-KIFC1 polyclonal antibody. The anti-KIFC1 polyclonal antibody recognized a 78 kDa band indicative of KIFC1 (Fig. 7, upper panel). The control was treated with anti-*β*- actin polyclonal antibody (Fig. 7, lower panel). We found that KIFC1 protein expression occurs in *E. modestus* testis, heart, muscle, hepatopancreas, and gill.

### KIFC1 cellular transformations during the spermiogenesis in *E. modestus*

**Figure 8:**
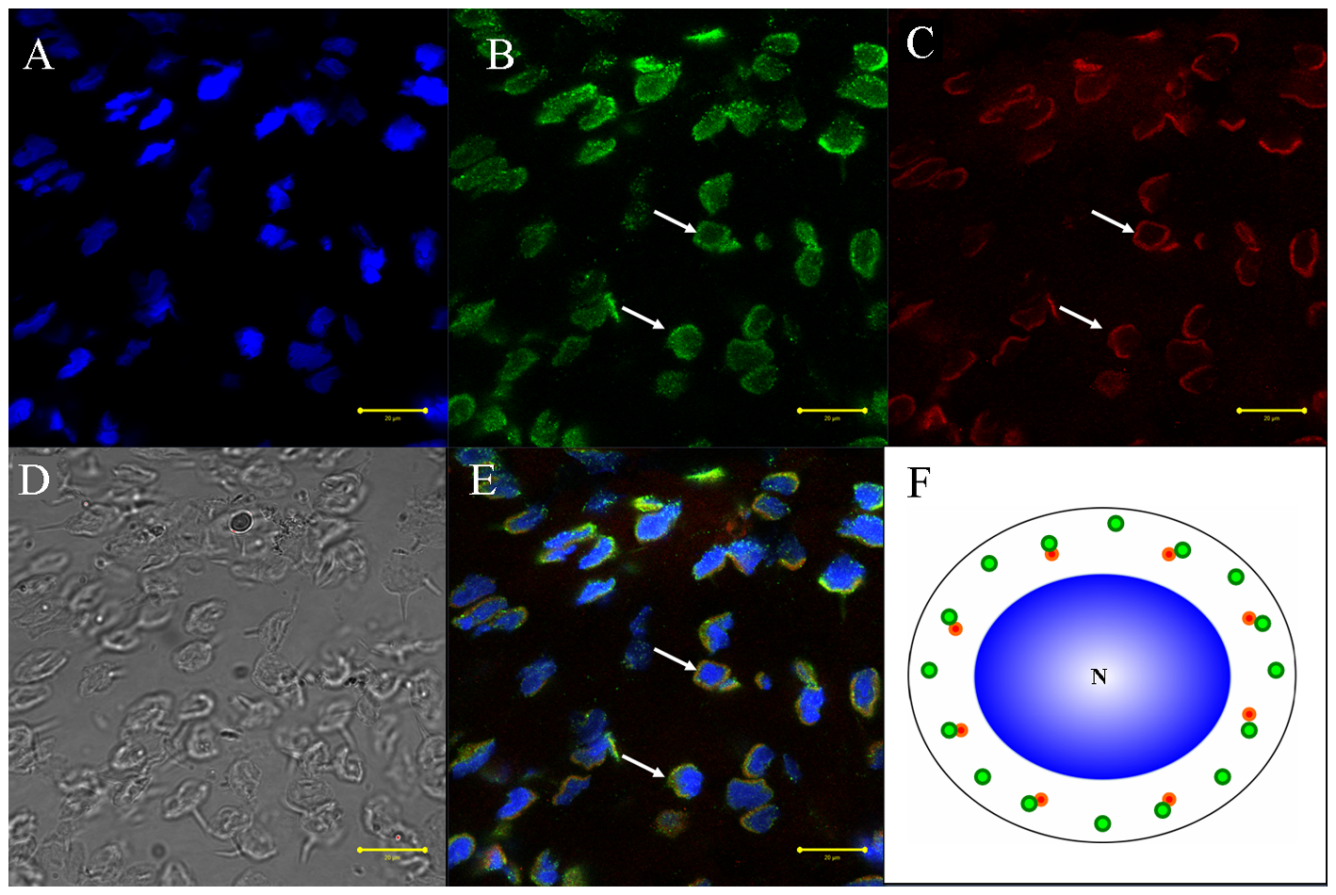
Immunofluorescent localization of tubulin and KIFC1 during the early stage of *E. modestus* spermiogenesis. (A) DAPI nuclear staining. (B) Tubulin staining. Tubulin (green staining; arrows) localizes in the cytoplasm and near the nuclear membrane. (C) KIFC1 staining. KIFC1 (red staining; arrows) also localizes in the cytoplasm and near the nuclear membrane. (D). Phase contrast microscopy. This figure shows the general morphological appearance of the spermatid during the early stage of spermiogenesis. (E) Merged immunofluorescent images. Tubulin (green staining; arrows) and KIFC1 (red staining; arrows) co-localize in the cytoplasm and near the nuclear membrane. Nucleus (blue staining). (F) Diagram. This diagram shows the distribution of tubulin (green dots) and KIFC1 (red dots) in the spermatid during the early stage of spermiogenesis. N: nucleus.

**Figure 9:**
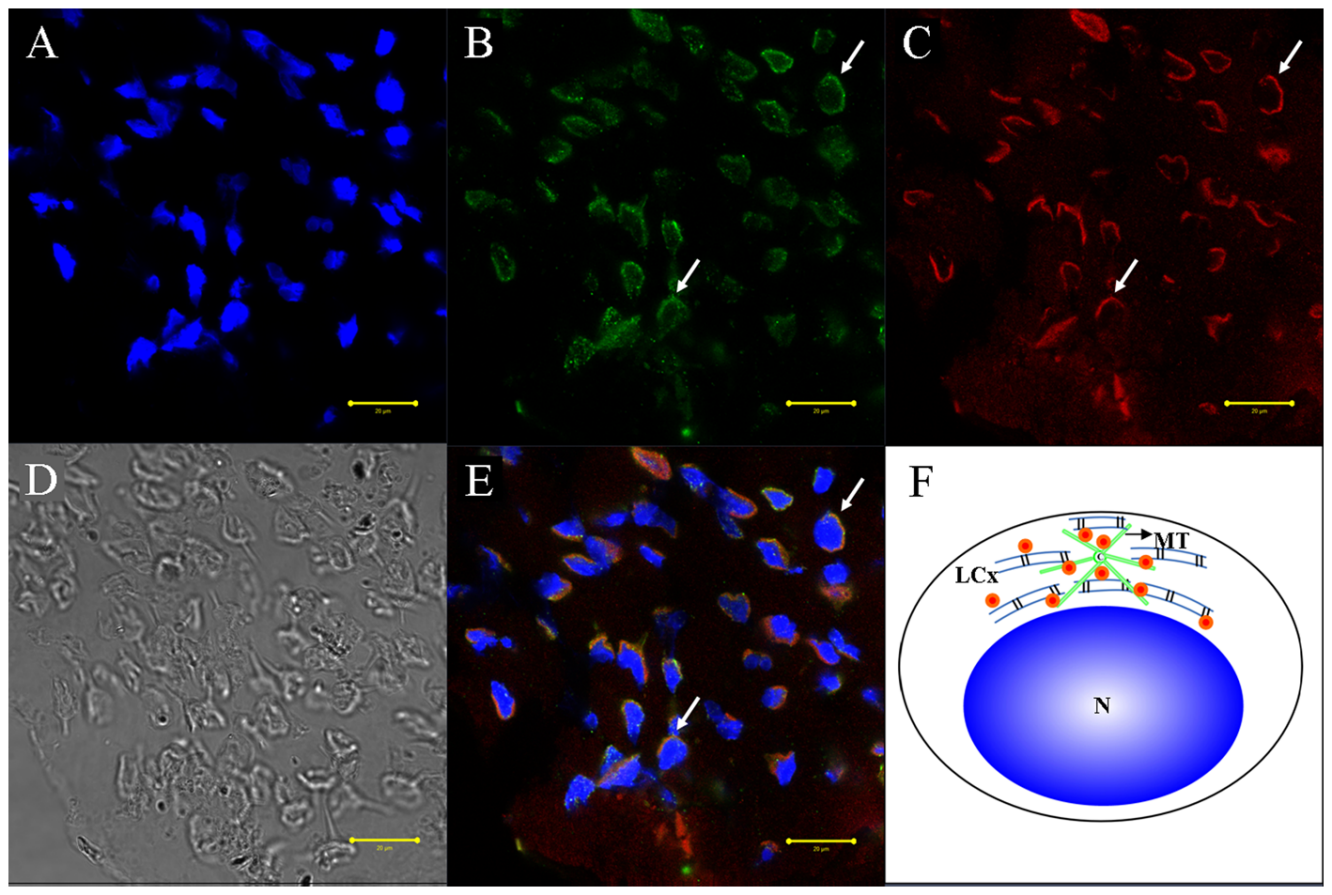
Immunofluorescent localization of tubulin and KIFC1 during the middle stage of *E. modestus* spermiogenesis. (A) DAPI nuclear staining. (B) Tubulin staining. Tubulin (green staining; arrows) localizes in sporadic areas within the lamellar complex (LCx). (C) KIFC1 staining. KIFC1 (red staining; arrows) localizes throughout the entire area of the LCx. (D) Phase contrast microscopy. This figure shows the general morphological appearance of the spermatid during the middle stage of spermiogenesis. (E) Merged immunofluorescent images. Tubulin (green staining; arrows) and KIFC1 (red staining; arrows) co-localize in relationship with the LCX. Nucleus (blue staining). (F) Diagram. This diagram shows the distribution of tubulin (green dots) and KIFC1 (red dots) in the spermatid during the middle stage of spermiogenesis. N: nucleus, LCx: lamellar complex, MT: microtubules, C: centriole.

**Figure 10:**
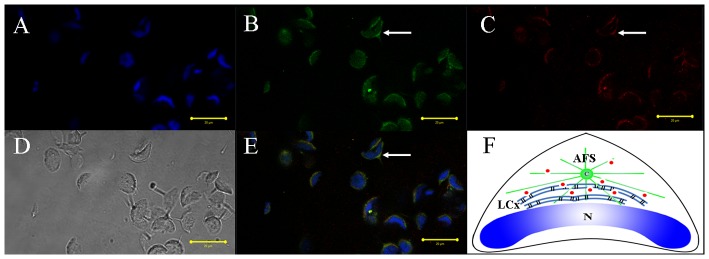
Immunofluorescent localization of tubulin and KIFC1 during the late stage of *E. modestus* spermiogenesis. (A) DAPI nuclear staining. (B) Tubulin staining. Tubulin (green staining; arrows) localizes specifically to the acroframosome (AFS). (C) KIFC1 staining. KIFC1 (red staining; arrows) localizes mainly to the proacrosome with some staining associated with the AFS. (D) Phase contrast microscopy. This figure shows the general morphological appearance of the spermatid during the late stage of spermiogenesis. (E) Merged immunofluorescent images. Tubulin (green staining; arrows) and KIFC1 (red staining; arrows) localization is shown. Nucleus (blue staining). (F) Diagram. This diagram shows the distribution of tubulin (green) and KIFC1 (red dots) in the spermatid during the late stage of spermiogenesis. N: nucleus, LCx: lamellar complex, AFS: acroframosome, C: centriole.

**Figure 11:**
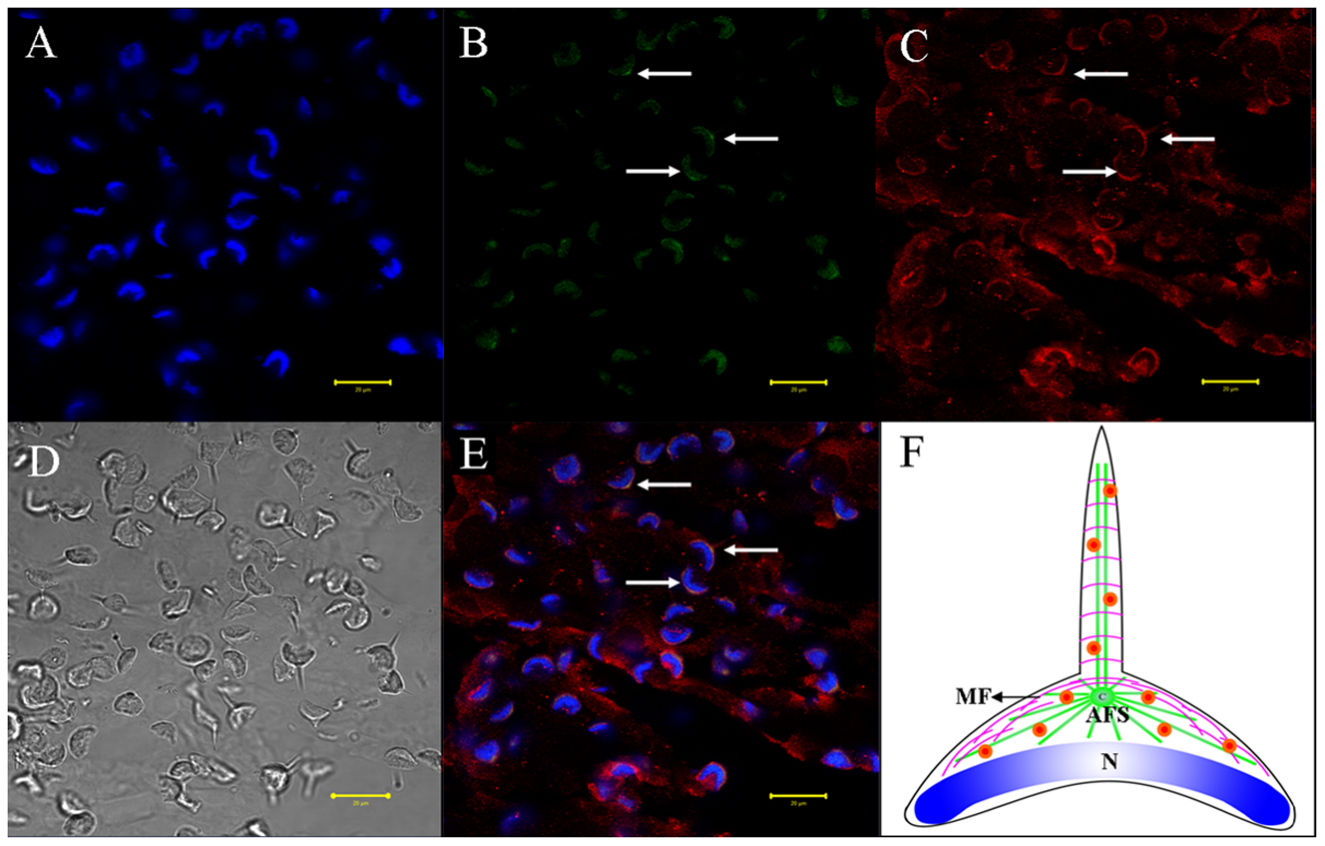
Immunofluorescent localization of tubulin and KIFC1 in mature *E. modestus* sperm. (A) DAPI nuclear staining. (B) Tubulin staining. Tubulin (green staining; arrows) localizes specifically to the center of the acrosome. (C) KIFC1 staining. KIFC1 (red staining; arrows) localizes throughout the whole acrosome including the AFS-derived spike (D) Phase contrast microscopy. This figure shows the general morphological appearance of the *E. modestus* mature sperm. (E) Merged immunofluorescent images. Tubulin (green staining; arrows) and KIFC1 (red staining; arrows) localization is shown. Nucleus (blue staining). (F) Diagram. This diagram shows the distribution of tubulin (green) and KIFC1 (red dots) in the *E. modestus* mature sperm. N: nucleus, MF: microfilaments AFS: acroframosome, C: centriole.

We used immunofluorescent localization of tubulin and KIFC1 in order to explore the role of microtubules and KIFC1 in cellular transformations during spermiogenesis in *E. modestus* (Fig. 8, 9, 10, 11). During the early stage of spermiogenesis, tubulin (Fig. 8B and E, arrows; 8F; green signal) and KIFC1 (Fig. 8C and E, arrows; 8F; red signal) localize in the cytoplasm and near the nuclear membrane. During the middle stage of spermiogenesis when the LCx forms, tubulin localizes in sporadic areas within the LCx (Fig. 9B and E, arrows; 9F). However, KIFC1 localizes throughout the entire area of the LCx (Fig. 9C and E, arrows; 9F). During the late stage of spermiogenesis when the AFS forms, tubulin localizes specifically to the AFS (Fig. 10B and E, arrows; 10F). However, KIFC1 localizes mainly to the proacrosome with some staining associated with the AFS (Fig. 10C and E, arrows; 10F). In the mature sperm, tubulin localizes primarily in the center of the acrosome (Fig. 11B and E, arrows; 11F). However, KIFC1 localizes throughout the whole acrosome including the AFS-derived spike (Fig. 11C and E, arrows; 11F).

### Manchette location during rat spermatogenesis

**Figure 12:**
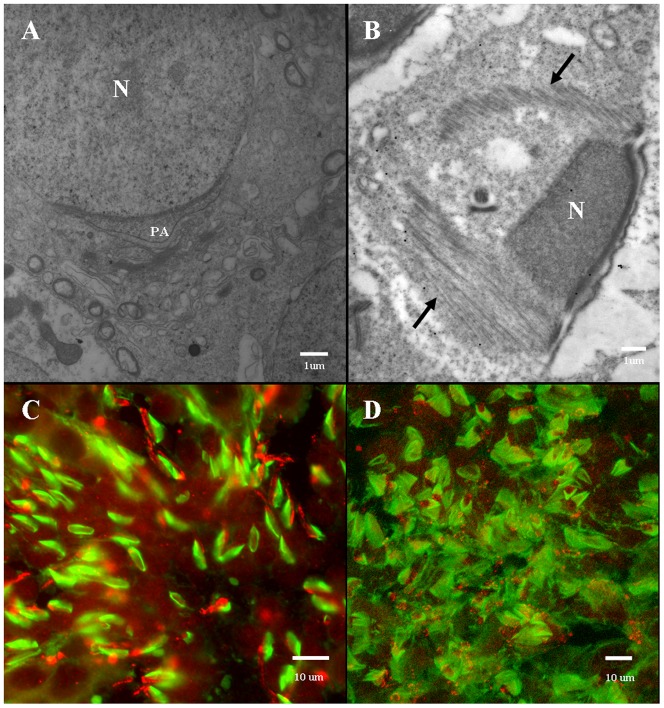
Morphological features of the manchette during rat spermatogenesis. (A,B) Electron microscopy. (A) This electron micrograph shows the absence of the manchette at the start of acrosome formation in rat spermatogenesis. N: nucleus, PA: proacrosome (B) This electron micrograph shows the presence of the manchette (arrows) during nuclear shaping in rat spermatogenesis. N: nucleus (C,D) Immunofluorescent staining. These immunofluorescent light micrographs show the localization of tubulin (green staining) and KIFC1 (red staining) in rat spermatids. The manchette appears to surround the nucleus in a skirt-shaped manner.

We used electron microscopy to demonstrate the distinction between the role of the manchette in rat spermatogenesis and the AFS in *E. modestus* spermatogenesis. We did not find proves which show that the manchette in rat spermatogenesis plays a role in acrosome formation (Fig. 12A), but rather plays a major role in nuclear shaping (Fig. 12B). Immunofluorescent staining of tubulin and KIFC1 showed a detached manchette that surrounds the nucleus in a skirt-like manner (Fig. 12C, D).

### KIFC1 transport substrates

**Figure 13:**
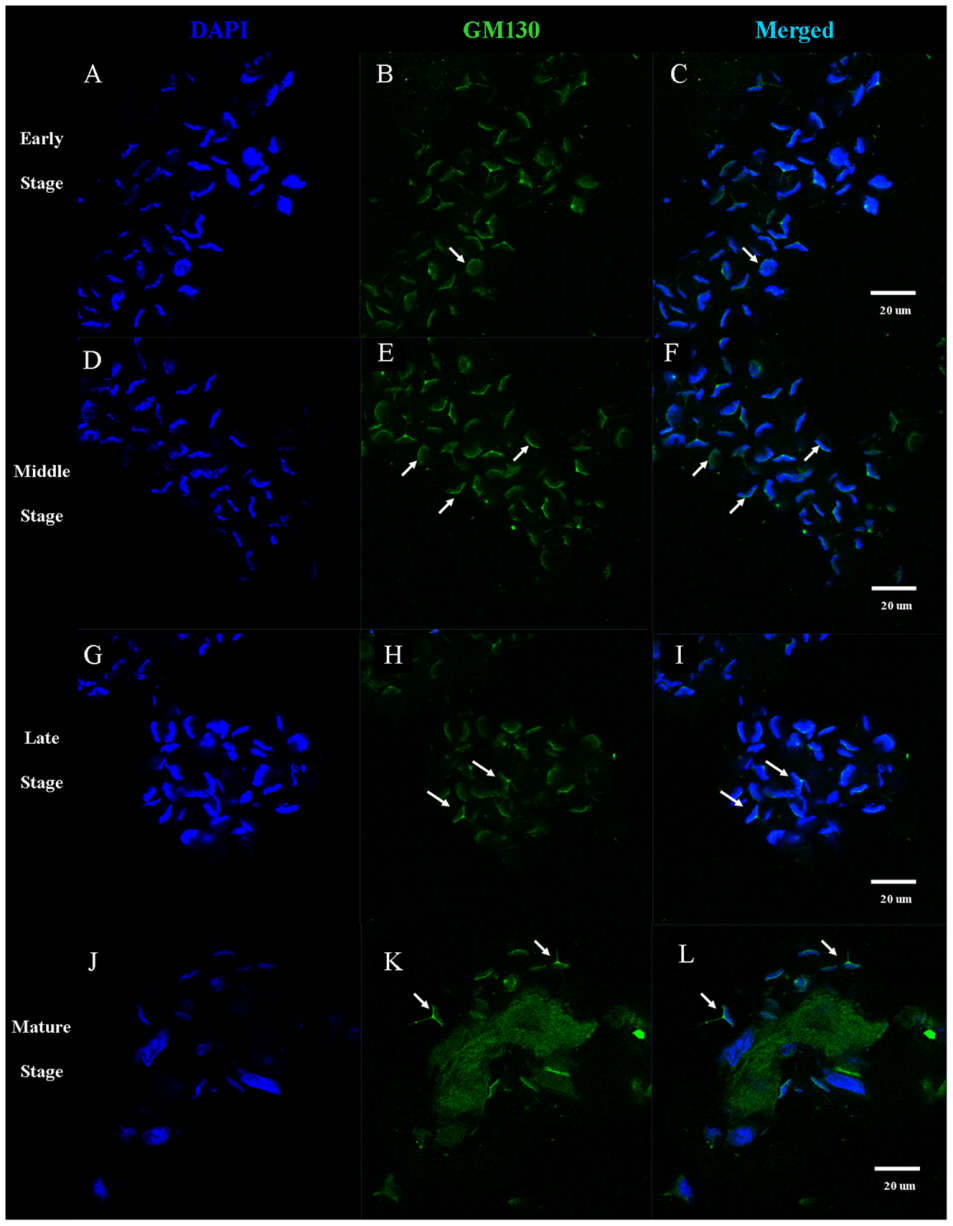
Immunofluorescent localization of GM130 (Golgi complex) during *E. modestus* spermiogenesis. (A–C) Early stage of spermiogenesis. (A) DAPI staining. (B) GM130 staining. GM130 (green staining; arrows) localizes in the cytoplasm near the periphery of the nucleus. (C) Merged immunofluorescent images. (D,E,F) Middle stage of spermiogenesis. (D) DAPI staining. (E) GM130 staining. GM130 (green staining; arrows) localizes near the LCx and along one side of the nucleus as the nucleus gradually deforms. (F) Merged immunofluorescent images. (G,H,I) Late stage of spermiogenesis. (G) DAPI staining. (H) GM130 staining. GM130 (green staining; arrows) localizes in the LCx and in the proacrosome near the AFS. (I) Merged immunofluorescent images. (J,K,L) Mature stage. (J) DAPI staining. (K) GM130 staining. GM130 (green staining; arrows) localizes in the acrosome. (L) Merged immunofluorescent images.

**Figure 14:**
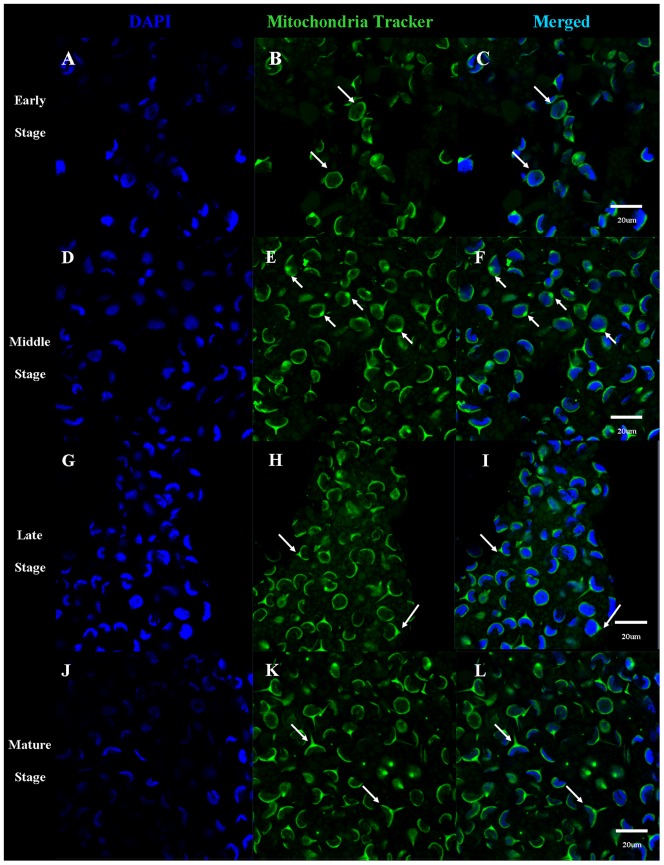
Immunofluorescent localization of MitoTracker (mitochondria) during *E. modestus* spermiogenesis. (A–C) Early stage of spermiogenesis. (A) DAPI staining. (B) MitoTracker staining. MitoTracker (green staining; arrows) localizes in the cytoplasm near the periphery of the nucleus. (C) Merged immunofluorescent images. (D,E,F) Middle stage of spermiogenesis. (D) DAPI staining. (E) MitoTracker staining. MitoTracker (green staining; arrows) localizes near the LCx and along one side of the nucleus as the nucleus gradually deforms. (F) Merged immunofluorescent images. (G,H,I) Late stage of spermiogenesis. (G) DAPI staining. (H) MitoTracker staining. MitoTracker (green staining; arrows) localizes in the LCx and in the proacrosome near the AFS. (I) Merged immunofluorescent images. (J,K,L) Mature stage. (J) DAPI staining. (K) MitoTracker staining. MitoTracker (green staining; arrows) localizes in the acrosome. (L) Merged immunofluorescent images.

**Figure 15:**
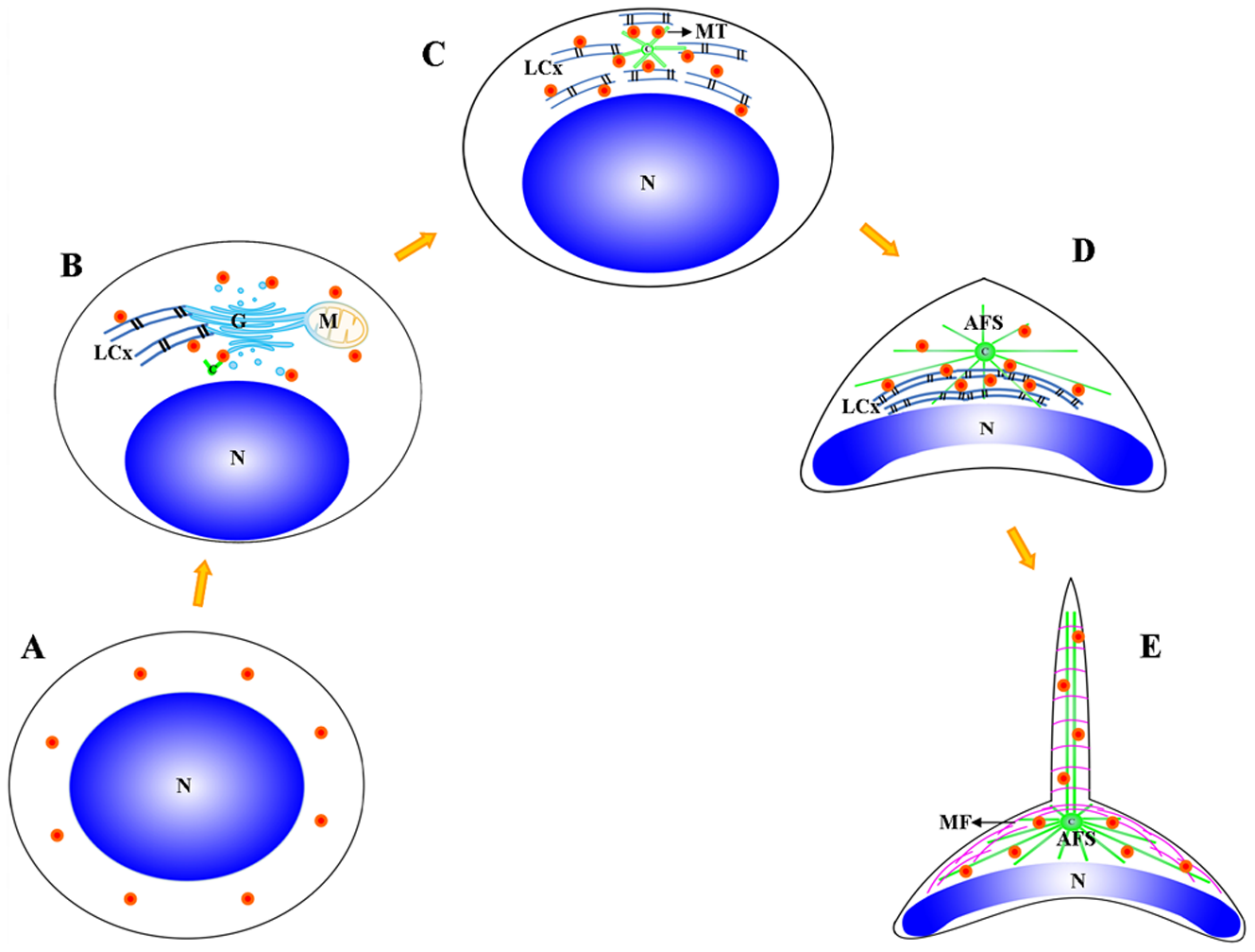
The distribution model of GM130 (Golgi complex) and MitoTracker (mitochondria) staining during *E. modestus* spermiogenesis. The GM130 and the MitoTracker staining patterns are concordant so they are both represented by orange dots. (A) Early stage of spermiogenesis. (A) Early stage of spermiogenesis. GM130 and MitoTracker localize in the cytoplasm near the periphery of the nucleus. (B) Middle stage of spermiogenesis. GM130 and MitoTracker localize near the LCx and along one side of the nucleus as the nucleus gradually deforms. (C,D) Late stage of spermiogenesis. GM130 and MitoTracker localize in the LCx and in the proacrosome near the AFS, (E) Mature stage. GM130 and MitoTracker localize in the acrosome. M: mitochondrion, N: nucleus, C: centriole, MT: microtubule, MF: microfilament, LCx: lamellar complex, AFS: acroframosome.

We used immunofluorescent localization of GM130 (a Golgi complex marker) and MitoTracker (a mitochondria marker) in order to identify KIFC1 transport substrates involved in acrosome formation (Fig. 13, 14, 15). During the early stage of spermiogenesis, GM130 localizes in the cytoplasm near the periphery of the nucleus (Fig. 13A–C: Fig. 15A). During the middle stage of spermiogenesis, GM130 localizes along one side of the nucleus as nucleus gradually deforms (Fig. 13D–F; Fig. 15B, C). During the late stage spermiogenesis, GM130 localizes in the LCx and in the proacrosome near the AFS (Fig. 13G–I; Fig 15D). In mature sperm, GM130 localizes in the acrosome (Fig. 13J–L; Fig. 15E). MitoTracker localizes in the same fashion as GM130 during the different stages of spermiogenesis in *E. modestus* (Fig. 14, 15).

### The audio video interactive of *E. modestus* spermatid

The audio video interactive shows the stereochemical morphology of *E. modestus* spermatids during the late stage of spermiogenesis (Video S1). The video shows a dish-shaped nucleus (blue) and the distribution of the microtubular structure AFS (green).

## Discussion

### The comparison of acroframosome and manchette

During mammalian spermatogenesis, the microtubular structure called the manchette appears in the developing spermatids and then disappears in the mature sperm [41]–[43]. The skirt-like manchette functions in nuclear shaping by acting as a scaffold via kinesin and dynein motor proteins in order to transform the nucleus from a spherical-shape to an oblong-shape [44]–[48]. In this regard, abnormal sperm heads and nuclear morphogenesis occur when the synthesis of tubulin (i.e., microtubules) is blocked [45]. A number of motor proteins are key players in spermatogenesis. During mammalian spermatogenesis, KIFC1 associates with the manchette and a complex containing nucleoporin NUP62 and facilitates nuclear shaping [26]. During spermatogenesis in *Octopus tankahkeei*, a KIFC1-like motor protein associates with the manchette and facilitates nuclear shaping [29]. In addition, another motor protein called dynein associates with the spermatid nuclear envelope during spermatogenesis in mammals [48]–[50].

During caridean shrimp spermatogenesis, we previously reported that a unique microtubular structure called the acroframosome (AFS) is formed which structure is similar to the manchette [37]. The everted umbrella-shaped AFS may play functions during acrosomal formation in the caridean shrimp, whereas manchette mainly functions in nuclear shaping in the rat. According to previous research in our lab, AFS may also contain microfilaments to maintain the spermatids flexibility, which is one of the main differences between AFS and manchette [37]. The AFS is a permanent structure found in both caridean shrimp spermatids and mature sperm, whereas the manchette is a transient structure found in only in mammalian spermatids.

### KIFC1 may participate in acrosomal shaping and biogenesis

A comparison of the similarity of *E. modestus* KIFC1 with different species and the phylogenetic tree illustrated that *E. modestus* KIFC1 remains relatively conservative which indicates that KIFC1 maintains its vital function throughout evolution. Furthermore, KIFC1 showed a relatively high expression in the *E. modestus* testis which adds further weight to the idea that KIFC1 plays a vital function in *E. modestus* spermatogenesis.

The identification of cDNA sequence revealed the existence of *kifc1* isogenous gene in the testis of *E. modestus*. Concurrently, the identification of *kifc1* gene may indicate its vital functions in testis. Multiple sequence alignment showed the conservative functional domains, such as ATP-binding domains and the microtubule-binding domain. Structural prediction reveals the KIFC1 of *E. modestus* mainly contained C-terminus, the stalk region and the N-terminal [5], [25], [28]. The putative C-terminus is the microtubule-binding domain formed a head which walking along the microtubules. The function of predicted stalk region is to form an extended coiled-coil stalk. And the predicted N-terminal is a divergent tail to carry different cargoes. The comparison of the similarity with different species and the phylogenetic tree illustrated that the KIFC1 of *E. modestus* kept the relative conservative which may important for the functions of KIFC1 during the evolution.

Analysis of *kifc1* mRNA and KIFC1 protein expression in various tissues showed that KIFC1 has relatively high expression in the testis. This result may indicate the indispensable function of KIFC1 during spermatogenesis in *E. modestus*. In addition, the KIFC1 also expressed in the other tested tissues, which may suggest the importance of motor protein KIFC1 in crustacean *E. modestus*. It is worth to mention that there is a different KIFC1 expression pattern in different tissue and organs between mouse and the caridean shrimp *E. modestus*. Zhang and Sperry (2004) described that the expression of KIFC1 protein was very weak in muscle and heart of mouse [51], while the western blot analysis of *E. modestus* tissues shows abundant expression in muscle and heart. This may suggest a significant expression pattern between mammalian and crustaceans. The functions of KIFC1 in different tissue and organs in caridean shrimp need to be further investigated.

The acrosome formation is one of the most important events of the spermiogenesis of the caridean shrimp. The everted-embrella-like acrosome of the caridean shrimp mature sperm is a unique structure. However, the complicated cellular motility mechanism of the acrosome formation of the caridean shrimp is still unknown. KIFC1 is a C-terminal kinesin motor, which was proved to play crucial roles during spermiogenesis in rat [25]. Based on the results of ISH and immunofluorescence in this study, the KIFC1 has a close relationship in the acrosome formation. The everted-embrella-like acrosome formation is depended on a microtubular structure AFS, which linked the temporary organelle lamellar complex (LCx) together.

*In situ* hybridization was performed to track the temporal and spatial expression pattern of *kifc1* mRNA in different stages during spermiogenesis of *E. modestus*, which shows *kifc1* mRNA locations and will indicate the expressive locations of KIFC1 protein and the potential functions of KIFC1 (Fig. 6). The previous studies expound that KIFC1 played an important role in acrosome formation in mammalian and crustacean [25]–[30]. The ISH results do show that *kifc1* mRNA was closely associated with acrosome biogenesis and nuclear shaping during spermiogenesis of *E. modestus* (Fig. 6). At the middle stage, *kifc1* mRNA signals are increased than the early stage and flurry of signals are concentrated on one side (Fig. 6C, D and J). At the late stage, the *kifc1* mRNA signals are visibly strong and highly centralize in acrosome cap and displayed dot-like signatures in the surface of dish-like nucleus (Fig. 6E, F and K). That is to say, *kifc1* mRNA signals are rapidly increased in the period of acrosome formation and nuclear shaping which implies the key role of *kifc1*. At the early and mature stages, the expression *kifc1* signals are dramatically less (Fig. 6A, B, G and H). It may provide more evidence of *kifc1* role in acrosome formation and nuclear shaping. In addition, it seems that the location of *kifc1* is closely associated with the temporary organelle LCx and the microtubular structure AFS during the spermiogenesis of *E. modestus*. This phenomenon may indicate the mechanism of *kifc1* as a motor protein participated in acrosome formation and nuclear shaping (Fig. 6I–L).

The immunofluorescence of KIFC1 and tubulin was performed to explore the role of KIFC1 in cellular transformations during spermatogenesis of *E. modestus*. Microtubes are consisted of tubulin, the gathered signals of tubulin stand for microtubule. The results of dynamic change of KIFC1 and tubulin in different stages obviously show that KIFC1 signals always distribute in the structure AFS, which linked the temporary organelle LCx together during spermiogenesis. From the early stage to the middle stage, with the formation of LCx and the emergence of AFS structure, KIFC1 signals are transfered to one side of the nucleus (Fig. 8 and 9). Microtubules are sporadically localized in the middle of LCx with dotted distribution (Fig. 8 and 9). At the late stage, the LCx is compressed and promoted the formation of everted-embrella-like acrosome (Fig. 10). The KIFC1 signals always appear in LCx and they overlap with microtubules (Fig. 10). These results may indicate an underlying mechanism that the formation of LCx is depended on the structure of AFS. KIFC1 transport cargoes, such as LCx, moving on the microtubules of AFS to facilitate the acrosomal morphogenesis.

**Figure 16:**
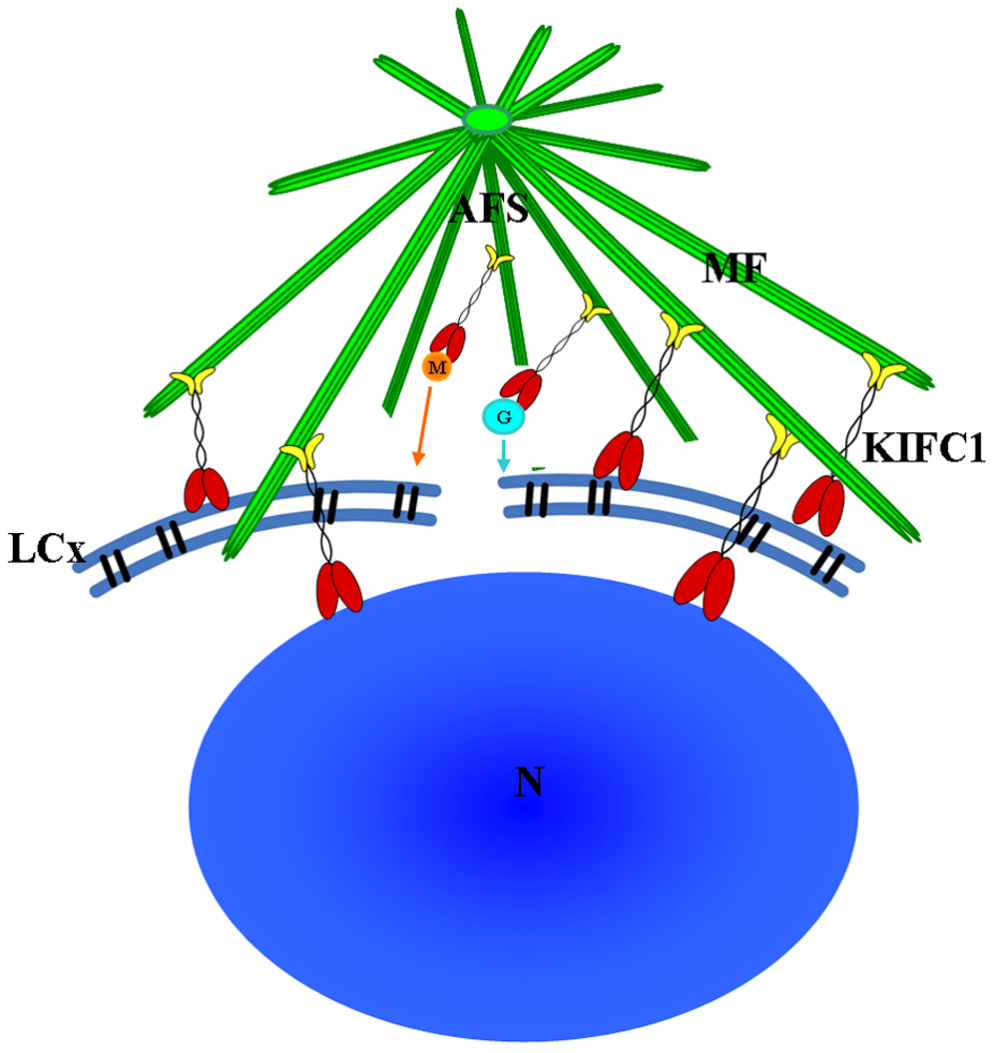
The mechanism of KIFC1 participation in acrosome formation during *E. modestus* spermiogenesis. T his model shows that KIFC1 moves along the AFS and transports Golgi complex vesicles, mitochondria, and probably other cellular components to fuse into the LCx. KIFC1 transports LCx components along the AFS in order to facilitate acrosome formation during *E. modestus* spermiogenesis. KIFC1 may also link to the nuclear envelope and participate in nuclear shaping. AFS: acroframosome, LCx: lamellar complex, MF: microfilament, M: mitochondrion, G: Golgi complex, N: nucleus.

The acrosomal infilling contains some organelle components, such as Golgi apparatus and mitochondria [34], [36]. As the temporary organelle LCx is the precursor of acrosome, the compositions of them are coincident [34], [36]. The immunofluorescence of GM130 and Mito-Tracker Green are to explore the role of KIFC1 in transportation of acrosomal infilling (Fig. 13, 14 and 16). In early spermatids, both GM130 and Mito-Tracker are equably distributed around the nucleus (Fig. 13B, Fig. 14B). However, with the formation of LCx, the GM130 and Mito-Tracker are almost gathered in a point of the spermatids, where is the AFS formed position in middle spermatids (Fig. 13E, Fig. 14E). Moreover, combining with the immunofluorescence of KIFC1 and microtubules, the signals of GM130 and Mito-Tracker have part overlapped position with them. In later spermatids, the phenomenon is even more pronounced. In the mature sperm, KIFC1, GM130 and Mito-Tracker are found in the acrosome (Fig. 13K, Fig. 14K). These results show a transported mechanism that KIFC1 transport organelles of dismissed protein as cargoes moving on the microtubules of AFS to facilitate the acrosomal formation during spermatogenesis in *E. modestus* (Fig. 16)

## Conclusion

KIFC1, the acroframosome, and the LCx all play a role in the formation of the everted umbrella-shape acrosome and nuclear shaping in caridean shrimp. The dynamic changes in tubulin and KIFC1 localization during different stages of spermiogenesis show that KIFC1 always distributes in the AFS which links to the LCx. This indicates that KIFC1 probably transports various cargoes along the microtubules of the AFS to facilitate acrosome formation and nuclear shaping. The transported cargoes include but are not limited to the Golgi complex and mitochondria.

This study provides a model to explore the molecular mechanisms of spermatogenesis in different crustacean species which will lead to further understanding of the fertilization mechanisms in crustaceans.

## Supporting Information

Video S1 The audio video interactive of *E. modestus* spermatid. The video shows the stereochemical morphology of *E. modestus* spermatids during the late stage of spermiogenesis. The video shows a dish-shaped nucleus (blue) and the distribution of the microtubular structure AFS (green).
(AVI)
